# Extending and encoding existing biological terminologies and datasets for use in the reasoned semantic web

**DOI:** 10.1186/2041-1480-3-6

**Published:** 2012-07-20

**Authors:** Soroush Samadian, Bruce McManus, Mark D Wilkinson

**Affiliations:** 1 UBC James Hogg Research Center, Institute for Heart Lung Health, St. Paul's Hospital, Room 166, Burrard Building 1081 Burrard Street, Vancouver, BC, Canada, V6Z 1Y6; 2 NCE CECR Center of Excellence for the Prevention of Organ Failure (PROOF Centre), St. Paul's Hospital, Room 166, Burrard Building 1081 Burrard Street, Vancouver, BC, Canada, V6Z 1Y6; 3 Centro de Biotecnología y Genómica de Plantas, Universidad Politécnica de Madrid, Madrid, Spain

## Abstract

****Background**:**

Clinical phenotypes and disease-risk stratification are most often determined through the direct observations of clinicians in conjunction with published standards and guidelines, where the clinical expert is the final arbiter of the patient’s classification. While this "human" approach is highly desirable in the context of personalized and optimal patient care, it is problematic in a healthcare research setting because the basis for the patient's classification is not transparent, and likely not reproducible from one clinical expert to another. This sits in opposition to the rigor required to execute, for example, Genome-wide association analyses and other high-throughput studies where a large number of variables are being compared to a complex disease phenotype. Most clinical classification systems and are not structured for automated classification, and similarly, clinical data is generally not represented in a form that lends itself to automated integration and interpretation. Here we apply Semantic Web technologies to the problem of automated, transparent interpretation of clinical data for use in high-throughput research environments, and explore migration-paths for existing data and legacy semantic standards.

****Results**:**

Using a dataset from a cardiovascular cohort collected two decades ago, we present a migration path - both for the terminologies/classification systems and the data - that enables rich automated clinical classification using well-established standards. This is achieved by establishing a simple and flexible core data model, which is combined with a layered ontological framework utilizing both logical reasoning and analytical algorithms to iteratively "lift" clinical data through increasingly complex layers of interpretation and classification. We compare our automated analysis to that of the clinical expert, and discrepancies are used to refine the ontological models, finally arriving at ontologies that mirror the expert opinion of the individual clinical researcher. Other discrepancies, however, could not be as easily modeled, and we evaluate what information we are lacking that would allow these discrepancies to be resolved in an automated manner.

**Conclusions:**

We demonstrate that the combination of semantically-explicit data, logically rigorous models of clinical guidelines, and publicly-accessible Semantic Web Services, can be used to execute automated, rigorous and reproducible clinical classifications with an accuracy approaching that of an expert. Discrepancies between the manual and automatic approaches reveal, as expected, that clinicians do not always rigorously follow established guidelines for classification; however, we demonstrate that "personalized" ontologies may represent a re-usable and transparent approach to modeling individual clinical expertise, leading to more reproducible science.

## **Background**

Terminologies and Nosologies have long been used by clinicians and clinical researchers as a means of more consistently annotating their observations. It is not surprising, then, that the emergence of the Semantic Web found fertile ground in the clinical and life science communities, and formal Semantic Web standards have been rapidly adopted by these communities to migrate existing annotation systems into these modern frameworks and syntaxes. While this largely syntactic migration is a useful exercise, in that it becomes possible to do simple reasoning over manual annotations, this simple migration does not enable the full power of modern semantic technologies to be applied to these important biomedical datasets. This is, in part, because these semantic resources continue to be used largely as controlled vocabularies rather than as rich descriptors for logical classification.

The Semantic Web languages Resource Description Framework (RDF) [1] and Web Ontology Language (OWL) [2] are the World Wide Web Consortium's recommended standards for semantically-explicit encoding of data and knowledge representation on the Semantic Web (respectively), and as such, these were the languages chosen for this study. Given the ability for RDF and OWL to be used to interpret, rather than simply annotate data, it would be useful to examine the migration path - both for the terminologies and the data - that enables such rich interpretive reasoning to be applied. How do we alter and/or extend existing terminologies such that they can be used to classify clinical data? What modifications to traditional data capture and representation must be made in order to make these data amenable to such logical inferences? Can we replace (or at a minimum, guide) expert clinical annotators in their interpretation of clinical data, and with what level of accuracy can this be achieved? In this report, we explore one such migration path, and discuss our observations and results, as well as the barriers and resulting manual-interventions that were employed to accomplish the goal of creating a reasoned environment for clinical data evaluation and interpretation. We base our exploration in a real-world use case, using clinical data collected and annotated 20 years ago in the context of a study of patient outcomes after various cardiovascular interventions.

Heart and Blood Vessel Diseases have a high rate of mortality and morbidity, and pose a significant disease-burden on healthcare systems worldwide. In such diseases, asymptomatic biological “diseases”, typically precede the clinical manifestation of symptomatic diseases. Most of the time, the development of biological disease into a symptomatic event can be significantly mitigated or prevented through a combination of medication and lifestyle changes. It is widely accepted that several risk factors including age, sex, high blood pressure, smoking, dyslipidemia, diabetes, obesity and inactivity are major factors for developing a variety of heart and blood vessel diseases [3]**.**

To assist with comparison of, and interpretation of, patient data, clinical researchers have developed guidelines for classifying patients phenotypically into various categories based on a wide variety of raw clinical measurements. For instance, Table 1 shows the American Heart Association (AHA) [4] guidelines for phenotypic classification of hypertension based on systolic and diastolic blood pressure observations. Although this classification system appears relatively straightforward, it is important to note that this represents only one of a number of different classification systems for the same phenotypic phenomenon (systemic hypertension), some of which include the informal expert-opinion of the clinicians themselves. As such, the same patient clinical observations might be categorized as “hypertensive” using one standard but categorized as “normal” using a different standard. This leads to problems when attempting to compare and integrate patient data between studies or even between different clinicians/centers in the same study, particularly when the annotation (“normal” *versus* “hypertensive”) is published in the dataset *in lieu* of the primary clinical measurements. To complicate matters further, health sciences communities continuously modify and update their guidelines in the light of new biomedical knowledge. For example, Global Initiative for Chronic Obstructive Lung Disease (GOLD) was comprehensively updated in 2006 which lead to different criteria for phenotypic classification with respect to previous years [5]. As such, even data from the same institution may be subject to slightly different interpretations over time. These interpretations become encoded in published datasets and, unfortunately, it is rare for the standards under which an interpretation was made to be rigorously recorded together with that interpretation. This issue leads to potentially erroneous re-interpretation of data, particularly when integrating data over long periods of time, or between disparate institutions. The emergence and uptake of Semantic Web technologies such as OWL and RDF by the Life Sciences, and the ability to use these technologies to enable dynamic classification of data, provides exciting opportunities for exploring novel ways to evaluate the feasibility of doing such clinical annotation dynamically.

**Table 1:** **American Heart Association classification for systolic and diastolic blood pressure **[4]

**Classification**	**Systolic pressure**		**Diastolic pressure**	
	**mmHg**	**kPa**	**mmHg**	**kPa**
Normal	90-119	12-15.9	60-79	8.0-10.5
Pre-hypertension	120-139	16.0-18.5	80-89	10.7-11.9
Stage 1	140-159	18.7-21.2	90-99	12.0-13.2
Stage 2	≥160	≥21.3	≥100	≥13.3
Isolated systolic hypertension	≥140	≥18.7	<90	<12.0

In this largely methodological study we undertook to create an environment in which “legacy” clinical data and annotation terminologies are modified such that they can be used together to automate the dynamic "on-demand" analysis and logical classification of patients into various cardiovascular disease risk groups under a variety of clinical classification guidelines. Specifically, we undertook a data remodeling process, migrating data from traditional databases and spreadsheets into a graph-based data framework (RDF); we utilize OWL to extend the cardiovascular-specific portion of an existing clinical annotation system namely GALEN [6] such that it can be utilized as an interpretation layer over this patient data; we then created a series of analytical Web Services which will be used to execute the statistical analyses of patient data in cases where pure logical reasoning is insufficient for classification; and finally, we executed our automated analyses/classifications, and compared them to the manual annotations done by an expert cardiovascular clinician two decades prior. Any differences were then examined in detail to determine the source of the discrepancy, and we evaluate and discuss our ability to modify the interpretive layers to account for differences between the clinician's manually annotated data and the automated annotations.

## **Methods**

### **Datasets and data collection**

The dataset used for this experiment consists of clinical observations of a cardiovascular patient cohort collected from a number of hospitals in Nebraska, USA from the period from August 1986 to July 1989. A total number of 636 unique patients with a total of 1723 encounters were recorded. The database was originally collected as a part of a study comparing the cardiovascular disease risk-profile changes over a period of one year post procedure/surgery for patients undergoing Coronary Allograft Bypass Graft (CABG) versus those undergoing percutaneous coronary intervention (PCI). An individual's risk can be assessed using a number of available risk-prediction tools such as Framingham [7], and Reynolds Risk Scores[8], which incorporate information on established risk factors such as blood lipids, Blood Pressure, Body Mass Index, age, gender, and smoking status. In this dataset two risk-assessment schemes were used to annotate patient data: a binary risk score ("at risk", "not at risk") assigned to individual clinical observations such as blood pressure, and an overall cumulative risk score using the Framingham risk measurement (see results section). The clinical observations used in this analysis were as follows:

" Age, Gender, Height, Weight, Body Mass Index (BMI), Systolic Blood Pressure (SBP), Diastolic Blood Pressure (DBP) Glucose, Cholesterol, Low Density Lipoprotein (LDL), High Density Lipoprotein (HDL), Triglyceride (TG) "

As an exemplar, the first row of the data set is shown in Table 2. The intended meaning of acronyms for each column header (e.g., SBP for Systolic Blood Pressure) was confirmed with the clinician who owned the dataset. The table contains two types of data: clinical observations (un-shaded cells), and the clinician-assessed binary risk - 1 or 0 for "at risk" or "not at risk", respectively (shaded cells; e.g., HDL GR for High Density Lipoprotein Risk Grade). The final column (RISK GR) indicates the ternary overall risk assessment - 1 for low, 2 for moderate, and 3 for high risk - which the clinician indicated to us was based on the Framingham Risk Score algorithms.

**Table 2:** Part of the first row of dataset used in Microsoft excel sheet

**SBP**	**DBP**	**TOTALCHOL**	**HDL**	**TG**	**AGE**	**GENDER**	**HEIGHT**	**WEIGHT**	**TG GR**	**HDL GR**	**LDL GR**	**CHOL GR**	**BMI GR**	**DBP GR**	**SBP GR**	**RISK GR**
128	80.1	227	55	84	77	M	1.8288	78.1818	**0**	**0**	**0**	**1**	**0**	**0**	**0**	**1**

### **Overview of approach**

In 2005 we proposed a semantic data classification architecture in which raw clinical measurements would be "lifted" through increasingly conceptual/interpretive layers of ontologies in order to complete an analysis, evaluation, or query [9]. This would be achieved through a combination of logical reasoning over the data and ontologies, in parallel with the discovery of Web Services that aggregated and analyzed the data, thereby dynamically identifying individuals logically compliant with the ontological classes at each layer. This hybrid approach is necessary because (useful) OWL reasoning is limited to a decidable fragment of first-order logic - effectively, it is possible to define the conditions under which an individual would be a member of a particular set/category, and it is possible to infer through a series of logical statements about the data, whether those conditions exist for a particular data record. However, while it is possible to infer that particular data properties must exist as a logical consequence of the existence of other data properties, it is not possible to *derive* data through algorithmic calculations using OWL reasoning alone. For these cases, we have written and published a series of Semantic Web Services that consume clinical data, execute various algorithmic analyses on them, and then return the dataset with new, derived data properties attached. These derived properties can then be used by the OWL reasoner to further classify the clinical data and "lift" it into increasingly complex clinical phenotypic categories.

While our approach is not reliant on any additional technologies for its success, one of our secondary goals in undertaking this project was to demonstrate that certain frameworks and practices established by our group could be used, with very little effort, to automate this interaction between OWL models and analytical Web Services. This automation reduces the complexity of analysis and evaluation of clinical data for the end-user. While the iterative process of reasoning, identification of appropriate analytical algorithms, execution of those algorithms, re-integration of output data, and re-reasoning could be done entirely manually (as would be the current practice), automating the "semantic lifting" process is enabled by two recently published pieces of technology - Semantic Automated Discovery and Integration (SADI) and the Semantic Health And Research Environment (SHARE).

SADI is a set of best-practices for modeling Semantic Web services in the scientific domain [10]. It is designed to be used in conjunction with OWL ontologies to discover Web Services capable of generating the properties that comprise an OWL class definition. Those Services, once discovered, are invoked by simple HTTP POST of RDF-formatted data.

SHARE is a SADI client application that allows SADI services to be discovered during the process of SPARQL-DL query evaluation [11]. Effectively, SHARE augments OWL reasoners and SPARQL query engines with the ability to retrieve data dynamically generated from remote data sources at the time of query execution and reasoning. When an ontological concept is present in a SHARE query, it will exhaustively "decompose" that concept into its complete set of property restrictions, importing any additional ontological classes as necessary. Once "decomposed", it then utilizes SADI to discover and execute services capable of creating those properties based on any data SHARE already has in its database.

**Figure 1:**
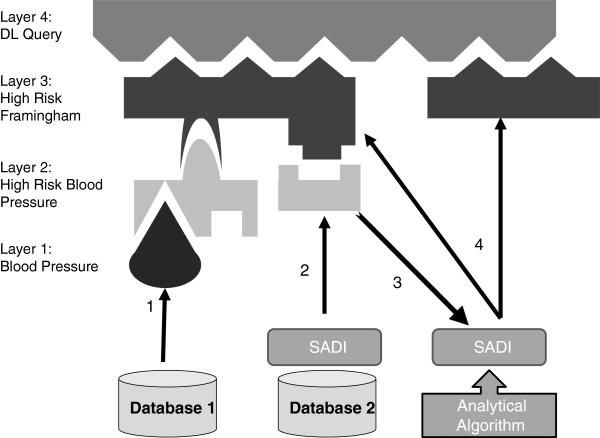
CardioSHARE architecture: increasingly complex ontological layers organize data into more abstract concept.

Figure 1 provides a diagrammatic representation of the "semantic lifting" process. By referring to an ontological concept in the SPARQL query (Layer 4 in the diagram), raw data is "lifted" through the ontological layers via an iterative process of reasoning, service discovery, and execution. This is our first attempt to deploy such an architecture over *bona fide* clinical data.

In order for this approach to be successful, we must first migrate the legacy data, and any legacy terminologies, into a more rigorous logical framework that is capable of being interpreted by OWL reasoners. We will now describe that process in detail.

### **Ontologies used**

#### ***Measurement unit ontology (MUO)***

While this study does not (conspicuously) take advantage of the semantic encoding of measurement units, we wish to nevertheless fully describe the process by which we transformed legacy data into a semantic framework. Since neither RDF nor OWL have a built-in method for representing units, it was necessary to select an approach to unit representation that would enable us, in future studies, to take advantage of their inherent semantics during query and reasoning.

MUO is a modular ontology specifically designed to represent units in a combinatorial fashion. MUO includes the definitions of the classes and properties conforming to the general design principles of upper-level Ontology DOLCE [12]. The ontology models mainly three disjoint entities: 1. Units of measurement; 2. Physical qualities that can be measured, and 3. Common prefixes for units of measurement. MUO also defines URIs for the most common units of measurement, “physical qualities” [13], and prefixes, which can be shared and reused in different domain ontologies. Every unit of measurement is attributed to a physical quality. Two types of units are generally dealt with in MUO: Base Units and Derived Units are defined as follows:

***Base Units*** are the units that are not derived from any other unit. Base units can be used to derive other units. The international System of Units (SI) It should be noted that even though ‘kilogram” is considered a base unit in SI, it is composed of kilo prefix plus base unit “gram”. In this sense, kilogram is an exception of a unit which is considered as base unit in SI system. However, to stick to our design schema, we considered “gram” as the base and defined kilogram as an extension of it,^a^ defines a number of independent base units such as meter (m) [14].

***Derived Units*** are the units obtained from combination of the base units of based units to represent “derived physical quantities” as defined by DOLCE [12]. In the formal representation of physical qualities and associated units, MUO defines property *muo:derivesFrom* to express the relationship between the derived unit and the units it is derived from. Derived Units are further divided into simple and complex derived units [14].

***Simple Derived Units*** are the units that are derived from exactly one base unit [14]. For instance, the millimeter (mm) can be derived from meter (m). These are units that can be defined by attaching a *Prefix* to base Units. MUO also recognizes a different type of base unit that although derived from exactly one base unit, has a different dimension. For instance *SquareMeter(m2).* For such cases another property called *muo:dimensionalSize* is added to account for the dimensionality differences.

***Complex Derived Units*** are the units that are derived from more than one base unit [14]. For instance consider Body Mass Index(BMI) which is a statistical measure which compares a person's weight and height. BMI is used to estimate a healthy body weight based on a person's height. BMI in International System of Units (SI) [15], is defined as follows [16]:

(1) $$\text{BIM}=\frac{\text{mass}\;\left(kg\right)}{{\left(\text{height}\left(m\right)\right)}^{2}}$$

BMI defined in this way, has the units **kg/m2** and this unit can be defined as follows using MUO:

:kilogram-per-meter-square rdf:type muo:ComplexDerivedUnit;

muo:derivesFrom ucum:kilogram;

muo:derivesFrom :meter-squared.

:meter-squared rdf:type muo:SimpleDerivedUnit;

muo:derivesFrom ucum:meter;

muo:dimensionalSize "2"^^xsd:float.

As shown above, MUO proposes a clear and convenient framework for defining new units of measurements in terms of existing ones, and this was used to derive any units required by our investigation not explicitly defined by the current version of the MUO.

#### ***GALEN***

The GALEN Common Reference Model (CRM) is a rich compositional ontology of the medical domain, covering anatomy, function, pathology, diseases, symptoms, drugs, and procedures [6]. It was developed by the Department of Computer Science at the University of Manchester [17]. It is available in both GRAIL [18] and OWL formalisms. The version used in this study is the latest OWL version available, dated August 2011 consisting of 2749 classes and 500 object properties. Several groups have investigated various aspects of the GALEN Ontology including expressivity, representation, and suitability for specific applications (e.g., [19]). Based on these studies, and our own investigation of the suitability of its terminological domain, we selected GALEN as our core Ontology describing cardiovascular concepts. In this paper we primarily focus on concepts in GALEN that are relevant to cardiovascular risk monitoring, and describe an approach for re-factoring and extending the cardiovascular-relevant classes of GALEN such that they can be used to automatically classify clinical data.

#### ***Semanticscience integrated ontology (SIO)***

The SemanticScience Integrated Ontology (SIO) is an effort to create a coherent formal ontology with rigorous attention to concrete and clearly-stated design patterns [20]. SIO takes the "realist" position in which things exist independently of conceptual or linguistic schemes, and firmly acknowledges that terms used in a discourse denotes one or more individuals or classes, for which the latter may have zero or more instances [21].

The choice of properties in development of any ontology is crucial and non-trivial [22]. The use of a minimal set of re-usable relations is essential in building consistent, interoperable and well-formed knowledge bases [23]. For instance, the following two OWL property constraints might be considered to describe the same data feature:

1. *Patient***hasAttribute** someValueFrom *SystolicBloodPressure*

2. *Patient***hasSystolicBloodPressure** someValueFrom *Attribute*

With respect to re-usability these two representations are considerably different. When designing ontologies to support logical reasoning, it is considered good-practice to encode the complexity of data in class definitions (statement #1) rather than through proliferation of properties (statement #2) [23]. The relationships defined by *SIO* are highly generic (e.g., "*has Attribute"*, defined by SIO's property *SIO_000008*), and this forces us, as the data modelers, to follow these good design patterns and formalize data-types through elaboration of ontological classes which are, whenever possible, distinct in their properties from all other ontological classes. We adhered to this design principle as closely as possible in this study.

Finally, SIO is extensively used by analytical tools exposed using SADI Semantic Web Services, and thus our adoption of SIO also allows us to more easily take advantage of existing analytical tools published through the SADI framework, as well as rapidly publish and integrate new tools as-needed for our study.

### **Unit representation in OWL-RDF**

When extracting datasets from disparate sites, particularly over international boundaries, it is not uncommon for the *de facto* unit of measurement to be different for any given clinical observation. Therefore, we must define a practical approach that allows clinical measurements to carry different units while not sacrificing interoperability. The lack of a standard approach to represent measurable quantities in RDF has led to a number of different configurations being used in different ontologies and RDF data repositories (see [14] for more information).

In our introduction of the MUO, we alluded to the fact that in context of the Semantic Web, representing physical quantities using ontologies is a non-trivial problem. RDF does not have any internal support for representing a literal value together with its unit of measure [14]. RDF literal nodes can represent numeric values, such as "120" or "141.5" without units, or value-unit pairs can be as strings of characters (e.g. “120 mmHg”); however, the "semantics" of the unit of measure is lost in both of these approaches, compromising our ability to accurately integrate datasets with heterogeneous measurements.

GALEN itself has a rather limited coverage of measurement units, and lacks a systematic framework to define new ones, or create composite units from basic ones. For instance, the GALEN concept *MilligramPerDeciLitre,* is defined as a subclass of the concepts *ConcentrationUnit,* but lacks any indication that this unit is composed of combination of two base units (gram and liter) and two prefixes (milli and deci). Similarly, SIO incorporates units from the Unit Ontology (UO) [24] in parallel with qualities from Phenotypic Quality Ontology (PATO) [25] for representing quantifiable measurements. However, like GALEN, SIO, UO, and PATO lack any formal framework for describing the relationship between related units, or defining new ones. Thus, in order to make use of such rich semantics in our analyses, we avoided the use of GALEN measurement units, preferring those defined by, or defined using, the MOU.

Nevertheless, though MUO does provide a method for defining the relationships between units and their derivatives, it does not provide a semantic framework for representing conversions between different units of the same "type" (e.g., metric and imperial weights). Since this was a potential problem in our analysis, and is a significant problem in science generally, we created a series of publicly-accessible Semantic Web Services capable of automatically detecting when unit-conflicts exist in an aggregated dataset, and automatically resolving those conflicts to whichever canonical measurement unit is desired.

### **Ontological mapping, extensions, and algorithmic services**

The set of OWL classes that are required to describe our dataset are as follows:

" Age, Sex, Mass, Height, BodyMassIndex, SystolicBloodPressure, DiastolicBloodPressure, BloodSugarConcentration, SerumCholesterolConcentration, SerumLDLCholesterolConcentration, SerumHDLCholesterolConcentration, SerumTriglycerideConcentration. "

We explored GALEN to search for the cardiovascular concepts listed above, and found it to be sufficiently comprehensive in terms of coverage of these concepts; however there were some minor differences in terminology between the labels in our dataset and GALEN terms. For example, the term *SerumHDLCholesterol* appears in GALEN, while the acronym *HDL* was used in our clinical dataset. Similarly the concept *Glucose* exists in GALEN while *BloodSugarConcentration* was the label applied to the (semantically) equivalent measurement in our clinical dataset. Such discrepancies were manually mapped based on consultation with expert clinicians*,* using their preferred labels. Our intent was to select the label/class-name that best semantically described the intended meaning of the concept; while we admit that this approach is somewhat arbitrary, we could think of no way to reliably automate these mappings.

The concept *Height* did not exist in GALEN, though the class *Length* did; to avoid over-loading the semantics of the existing GALEN class, we defined a new class *Height*, and made this a subclass (*owl:subClassOf*) of GALEN's *Length.*

Our proposed "layered" semantic framework requires us to identify concepts which are "core" (based on direct observations - Layer 1 of Figure 1), and concepts which are "derived" (based on calculations over core observations - Layer 2 and higher). For instance, the current lipid measurement protocols do not generally measure LDL particles directly but instead estimate them using the *Friedewald* equation [26]:

(2) H≈C−L−kT

where H is HDL cholesterol, L is LDL cholesterol, C is total cholesterol, T is triglycerides, and k is 0.20 if the quantities are measured in mg/dl and 0.45 if in mmol/l [27]. Thus, Triglycerides and Cholesterol are "core" measurements, while LDL is a "derived" measurement. Similarly, BMI is calculated from a relationship between height and mass, and would be considered "derived". We manually examined the protocols for obtaining the measurements in our dataset and consider the following GALEN classes to represent "core" measurements:

" Age, Sex, Mass, Height, SystolicBloodPressure, DiastolicBloodPressure, BloodSugarConcentration, SerumCholesterolConcentration, SerumHDLCholesterolConcentration, SerumTriglycerideConcentration. "

We henceforth will refer to these Classes as the "Grounding Classes" - classes whose members will be directly tied to the dataset through explicit declaration of a piece of data as being a member of that Class.

The OWL definition of each Grounding Class was created by extending the corresponding GALEN Class definition to include the defining features of the "Attribute" OWL Class from SIO; thus all members of these classes will (logically) be both GALEN individuals, and SIO Attributes. This involved adding axioms for the SIO properties *hasMeasurement, hasUnit* and *hasValue* to the GALEN class definitions*.*

The example below shows how GALEN class for Systolic Blood Pressure is extended using external classes and properties (the prefix before “:” shows the ontological namespace of each entity; the prefix "cardio" is the namespace used to indicate the ontological classes we have defined)

cardio:SystolicBloodPressure:

Galen:SystolicBloodPressure ***and***

*(*sio:hasMeasurement ***some***

cardio:pressuremeasurement*)*

cardio:pressuremeasurement:

sio:measurement ***and***

(sio:hasUnit ***some***

“muo:unit of pressure” ***and***

hasValue ***some*** Literal)

The remainder of the measurements in our clinical dataset are "derived", based on calculations performed over the core measurements, and their corresponding "Derivative Classes" in GALEN are:

" SerumLDLCholesterolConcentration BodyMassIndex "

The class definition for these was generated using the same approach as for the Grounding Classes; however, since members of these Derivative Classes can only be determined through algorithmic analysis of 'core' measurements, we also created a set of SADI Semantic Web Services that expose the necessary algorithms, consuming members of the relevant Grounding Classes, and generating members of the Derivative Classes in response. Thus, data from Layer 1 can be "raised" into Layer 2 Classes (and above) through invocation of these algorithmic services.

### **Refactoring the legacy dataset**

#### ***Assumptions about data collection and measurements***

Since the exact protocols describing *how* the clinical observations were made were not available, we made the assumption that they were derived from the most common measurement protocols. For example, for blood pressure measurements, we assumed that the measurements were made in a clinical setting (as opposed to casual home monitoring), using conventional mercury manometers applied on the left arm. The units used for each measurement were not explicitly stated in the datasets (Table 2) itself, so we made a best-guess based on the range of the measurement values and confirmed those with clinical experts. The units used to represent measurements are shown below.

***Height****: meter*

***Weight****: kilogram*

***BodyMassIndex****: kilogram per square meter*

***SystolicBloodPressure****,****DiastolicBloodPressure****: millimeter of mercury column*

***SerumHDLCholesterolConcentration****,****SerumLDLCholesterolConcentration****,****SerumTriglycerideConcentration****,****SerumCholesterolConcentration****,****BloodSugarConcentration****: milligram per deciliter*

#### ***Data schema***

**Figure 2:**
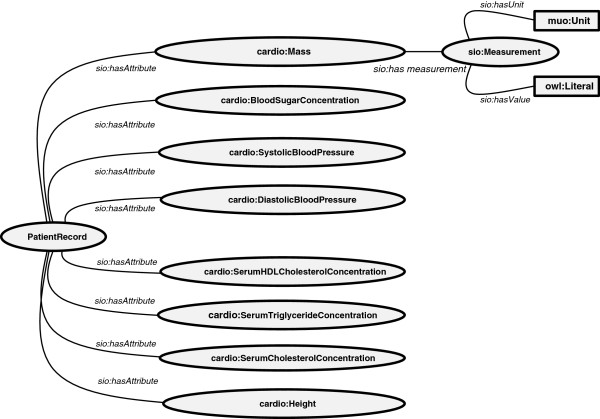
**Data schema using concepts in legacy ontologies.** The additional features shown on the Mass class are present on all classes in that row, but are hidden to improve readability.

Our primary objective in designing an ontological model to represent the clinical data was to support dynamic re-interpretation of that data under a variety of different hypothetical scenarios (e.g., re-interpretation as analytical or classification standards change over time). Importantly, it was not our intention to design a data model with sufficient complexity to represent every aspect of a clinical record; rather we were focused on modeling individual clinical measurements in a way that would allow them to be automatically analyzed and interpreted. Constraining ourselves to modeling only this small aspect of the clinical record should, we believe, allow existing comprehensive clinical record models to be easily adapted to the framework we propose here. Figure 2 shows the schematic view of the data model, described as follows:

1. We defined a class "*PatientRecord*", as a subclass of the SIO "*record*" class. *PatientRecord* will include all of the observations about a patient, keeping in-mind that we considered each patient-encounter to be a different patient record for the purposes of this study (i.e., the *longitudinality* of the data was not considered).

2. Patient clinical observations were divided into Grounding Classes and Derived Classes as described above, and were modeled as owl:Individuals of these classes, with the corresponding unit and value attached by the SIO hasUnit and hasValue properties.

3. Each resulting Grounding Class member was attached as an attribute of the *PatientRecord* using the *hasAttribute* property from SIO.

Finally, using MUO methodologies (described above), we defined the units *kilogram, kilogram-per-meter-squared, millimeter-of mercury-column, milli-gram-per-deci-liter,* and *milli-mole-per-liter*, which were used for various individual studies as described in the Results section.

### **Approach to binary patient classification (“at risk” versus “not at risk”)**

In our dataset the clinical researchers used a binary system to classify patients as being "at risk" or "not at risk" based on each of the following measures: Blood Pressure, Cholesterol, HDL, LDL, Triglycerides, and BMI. Thus, we created OWL classes representing each of these categories - for example, "HighRiskSBPRecord" to represent patient records reflecting a high-risk score with respect to Systolic Blood Pressure, and "LowRiskSBPRecord" to represent patient records reflecting a low-risk score with respect to Systolic Blood Pressure. These categories would then be used in SHARE SPARQL queries to trigger data "lifting", and to compare the result of the resulting automated categorization of patient records with the expert annotation of the clinical researchers two decades ago.

Working through one example in detail - Table 3 shows the American Heart Association's classification of systolic and diastolic blood pressure values. Although they indicate five different ranges (Normal, Prehypertension, Stage 1 hypertension, etc.) the clinical researchers who generated our dataset had only two categories - "at risk", and "not at risk". Through discussions with the researchers, they indicated that they considered Normal and Prehypertension to be "not at risk" (in Table 3) and all other categories to be "at risk". In Tables 3 through 6 the light shaded area represents “low risk” whereas the dark shaded area represents the “high risk” groups as defined by the guidelines. As such, we modeled an ontological class "HighRiskSBPRecord" in OWL as follows:

HighRiskSBPRecord =

cardio:PatientRecord ***and***

(sio:has Attribute ***some***

(cardio:SystolicBloodPressure ***and*** sio:hasMeasurement ***some***

(sio:Measurement ***and***

(sio:hasUnit ***value*** cardio:milli-meter-of-mercury-column) ***and***

(sio:hasValue ***some*** double[> = "140.0"^^double]))))

**Table 3:** **American Heart Association classification for systolic and diastolic blood pressure **[4]

**Classification**	**Systolic pressure**		**Diastolic pressure**	
	**mmHg**	**kPa**	**mmHg**	**kPa**
Normal	90-119	12-15.9	60-79	8.0-10.5
Pre-hypertension	120-139	16.0-18.5	80-89	10.7-11.9
Stage 1	140-159	18.7-21.2	90-99	12.0-13.2
Stage 2	≥160	≥21.3	≥100	≥13.3
Isolated systolic hypertension	≥140	≥18.7	<90	<12.0

In Tables 3 through 6 the light shaded area represents “low risk” whereas the dark shaded area represents the “high risk” groups as defined by the guidelines.

In a somewhat different scenario, Table 4 shows the American Association risk stratification for cholesterol, HDL and Triglycerides, each of which has three categories - high, medium, and low - compared to our clinician's binary categorization of high and low. As above, we attempted to create OWL classes to model these risks; however, in this case we had no guidance from the clinician as to what to do with intermediate measurements, as their original policy had not been recorded. As such, in our initial (somewhat trivial) analysis, we defined "high risk" and "low risk" records as being congruent with the high and low risk categories of the official guidelines, and ignored all data in the intermediate category. We describe how we modified these models, and our ability to determine the actual clinician's risk threshold, in the Results section.

**Table 4:** **American Heart Association classification for cholesterol, HDL, and triglycerides **[28,29]

	**Level (mg/dl)**	**Level (mmol/L)**	**Interpretation**
**Cholesterol**	<200	<5	Desirable level corresponding to lower risk
	200-240	5.2-6.2	Borderline high risk
	>240	>6.2	High risk
**HDL**	<40 for men, <50 for women	<1.03	Low HDL cholesterol, heightened risk
	40-59	1.03-1.55	Medium HDL level
	>60	>1.55	High HDL level, optimal condition
**Triglyceride**	<150	<1.69	Normal Range: low risk
	150-199	1.70-2.25	Borderline high
	200-499	2.26-5.65	High
	>500	>5.65	Very high: high risk

Modeling BMI and LDL risks were slightly more complex, since these two measurements are derived by algorithmic analysis of one or more 'core' measurements. BMI is calculated using a person’s weight and height (Equation 1) [15], and the guidelines were modeled in OWL following the American Heart Association guidelines in Table 5. The resulting OWL classes representing BMI and HighRiskBMI measurements respectively were as follows:

cardio:BodyMassIndex =

galen:BodyMassIndex ***and***

(sio:hasMeasurement ***some*** cardio:measurement)

cardio:measurement =

sio:measurement ***and***

*(*sio:hasUnit ***some*** cardio:UnitOfAreaDensity ***and***

sio:hasValue ***some*** Literal*)*

HighRiskBMI=

PatientRecord ***and***

(sio:hasAttribute ***some***

(cardio:BodyMassIndex ***and*** sio:hasMeasurement ***some***

(sio:Measurement ***and***

(sio:hasUnit ***value*** cardio:kilogram-per-meter-squared) ***and***

(sio:hasValue ***some*** double[> = 25.0]))))

**Table 5:** **American Heart Association classification for BMI **[30]

**BMI (kg/m**^**2**^**)**	**Category**
Below 18.5	Underweight
18.5 to 24.9	Healthy weight
25.0 to 29.9	Overweight
30 to 39.9	Obese
40 and above	Morbidly obese

**Figure 3:**
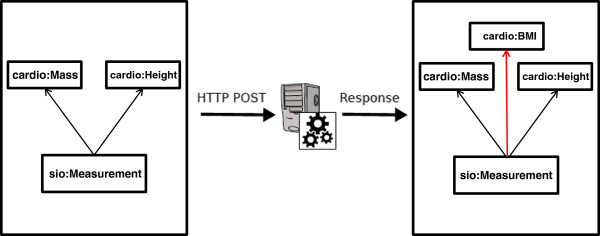
**The schematic diagram of the SADI web service interface to the BMI calculation service.** The property-restriction imposed on the output, when detected by SHARE, triggers the discovery and invocation of the Service that attaches the BMI class with appropriate units and value properties attached to it.

The schematic diagram of the SADI Web Service interface for BMI calculation is shown in Figure 3. The input and output of the Service is as follows (sample data, and instructions on how to send this data to the SADI service, are provided in the Supplementary Information [27]):

*Input:*

(sio:hasAttribute ***some*** cardio:Height) ***and***

(sio:hasAttribute ***some*** cardio:Mass)

*Output:*

sio:hasAttribute ***some***

(cardio:BodyMassIndex ***and***

(sio:hasMeasurement ***some*** ( sio:hasMeasurement ***and***

(sio:hasUnit ***value*** cardio:kilogram-per-meter-squared ***and***

(sio:hasValue ***some*** Literal)))

Subsequently, we calculated LDL in a similar fashion using SADI-compliant Semantic Web Services. . LDL is calculated based on HDL measurements via the *Friedewald* equation (Equation 2 [26]) and the guidelines were modeled in OWL following the guidelines in Table 6. Note that the *Friedewald* equation includes a constant that is sensitive to the units HDL is measured in; however since we are explicitly declaring and automatically converting units, this service is able to automatically determine which is the correct constant to use in every case.

**Table 6:** **LDL guidelines **[29]

**Level (mg/dl)**	**Level (mmol/L)**	**Interpretation**
<129	<3.3	Desirable level
130-159	3.3-4.1	Borderline high risk
>160	>4.1	High risk

### **Approach to ternary risk assessments**

In addition to the somewhat trivial binary risk assessments described above, we wish to determine whether more complex clinical phenotype and risk classifications can be automated using the same infrastructure. For example, some clinicians are more interested in estimating the probability of a patient developing a certain type of cardiovascular disease within a specific period of time. Researchers have developed a variety of algorithms for estimating a patient’s statistical probability of death (from cardiovascular disease) or of developing a variety of cardiovascular diseases, with one of the most widely adopted being the Framingham Risk Scores [8]. There are a number of different Framingham Risk Scores centered around different cardiovascular diseases (e.g., Congestive Heart Failure versus Atrial Fibrillation), the period of time under which the risk assessment is calculated (e.g., 5 year versus 10 year risk), and the precise Framingham standard used. For instance, the same patient clinical observations might be categorized as “high risk” using Canadian Standards, but categorized as “medium –high risk” using American or European Standards.

To test our ability to automatically classify patients into complex risk-stratification models such as Framingham, we created OWL models of the Framingham Risk Scores for General Cardiovascular Disease in Men [31]. Table 7 shows the scoring framework proposed by the Framingham study to calculate the estimated risk score for General Cardiovascular Disease in men based on the mean values for clinical observations. Similar tables exist for women and other cardiovascular diseases such as Arterial Fibrillation, Congestive Heart Failure, Coronary Heart Disease, General Cardiovascular Disease, Hard Coronary Heart Disease, Intermittent Claudication, Recurring Coronary Heart Disease, Stroke After Atrial Fibrillation.

**Table 7:** **Estimated risk of general cardiovascular disease in men **[31]

**Points**	**Age, y**	**HDL**	**Total cholesterol**	**SBP not treated**	**SBP treated**	**Smoker**	**Diabetic**
**−2**		60+		<120			
**−1**		50-59					
**0**	30-34	45-49	<160	120-129	<120	No	No
**1**		35-44	160-199	130-139			
**2**	35-39	<35	200-239	140-159	120-129		
**3**			240-279	160+	130-139		No
**4**			280+		140-159	Yes	
**5**	40-44				160+		
**6**	45-49						
**7**							
**8**	50-54						
**9**							
**10**	55-59						
**11**	60-64						
**12**	65-69						
**13**							
**14**	70-74						
**15**	75+						

In our dataset, clinician had annotated the records with three scores: “high risk”, “low risk” and “moderate risk”. For this study, we only considered the records of male patients and records with no missing values for the various observations required to make a risk evaluation.

The conventional classification used in Canadian health care system is based on three levels of quantization (0–9: low Risk, 10–19: Medium risk, > = 20: High risk) over the accumulated individual risk score (Table 8).

**Table 8:** **10-year risk for general CVD by total Framingham Risk Score **[31]

**Total points**	**10-year risk**
<9	<1%
9	1%
10	1%
11	1%
12	1%
13	2%
14	2%
15	3%
16	4%
17	5%
18	6%
19	8%
20	11%
21	14%
22	17%
23	22%
24	27%
25 or more	≥30%

The input and output classes for SADI web service to calculate the Framingham Risk Score are defined as follows:

***Input****:*

*PatientRecord****and***

*(sio:hasAttribute****some****cardio:Age)****and***

*(sio:hasAttribute****some****cardio:*

*SerumCholesterolConcentration)****and***

*(sio:hasAttribute****some****cardio:*

*SerumHDLCholesterolConcentration)****and***

*(sio:hasAttribute****some****cardio:SystolicBloodPressure*)

*Output:*

*sio:hasAttribute****some****(GeneralCVDFraminghamRiskScore****and***

(sio:hasValue some Literal))

Since an OWL class representing Risk Score did not exist in any of the Ontologies we were using, we defined a class named RiskScore and a second, GeneralCVDFraminghamRiskScore, which is a subclass of the former.

## **Results**

### **Evaluation of automated binary risk classification**

**Figure 4:**
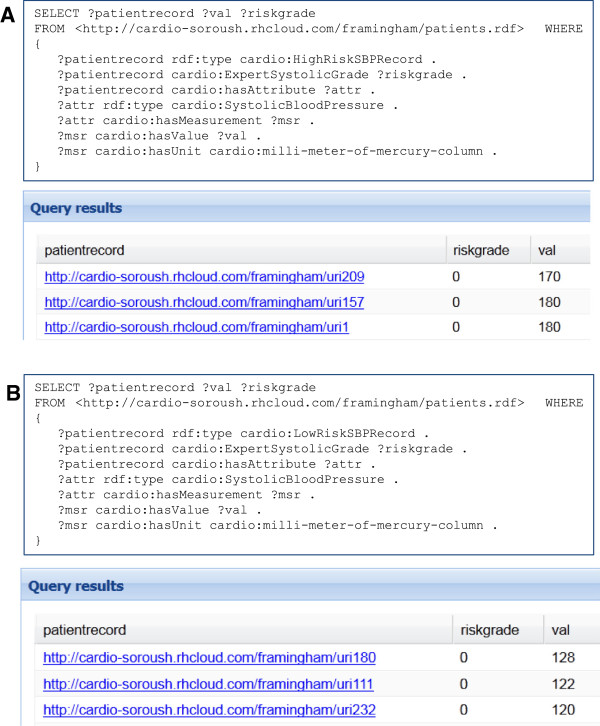
**SPARQL queries (Prefixes not shown) followed by a small snapshot of the results for automatic classification of patients into “high risk” (A) and “low risk” (B) for Systolic Blood Pressure.** Note that, because of unit conversion layer, the units used to model the guideline may or may not be the same as the unit used to model clinical data.

Evaluation of our ability to dynamically reproduce the original clinical classifications, using the approaches described above, was undertaken as follows: In the dataset, when the clinician had indicated the patient was "at risk" for a given type of observation, this was represented as a numeric "1", while if they indicated the patient was not at risk, we represented this as a numeric "0". We then used our "HighRisk" and "LowRisk" OWL Classes in SPARQL queries, calling-up the clinician-annotated numerical score in the same query. For each HighRisk query, we would expect the clinicians score to be "1" in all cases if our automated analysis is functioning correctly, and should be "0" in all cases for the LowRisk queries. Figure 4 shows two queries for SBP measurements and their clinician-assigned risk grade, together with a screen-shot of the abbreviated output for each query. If the system is calculating risk correctly, then all results of the query for high risk (Figure 4A) should be assigned a score of "1" by the clinician, and similarly the results of the query for low risk (Figure 4B) should be assigned a score of "0". Similar queries were issued for DBP, Chol, HDL, TG, and BMI attributes. Table 9 shows the comparison between manual and automatic risk classification for all attributes in the dataset. In most cases, our automated analysis of the data was entirely concordant with the expert annotations of the clinician; however, there were several cases of discrepancy as discussed in the next section. More detailed query/result pairs, plus before/after categorization data for all clinical observations can be found as supplementary material at [27].

**Table 9:** Comparison between manual and automatic binary risk classifications

	**True positive rate “at risk” %**	**False positive rate”at risk” %**
**SBP**	100	0
**DBP**	100	0
**CHOL**	92.6	0
**HDL**	100	56.5
**TG**	100	8.5
**BMI**	100	18.8
**LDL**	100	0

Correctness represents the degree of fidelity of automatic classification to that of the expert.

### **Discrepancies between automated and expert binary classifications**

#### ***Systolic and diastolic blood pressure risks***

Classifying patients as being “high risk” or “low risk” based on blood pressure was consistent with manual curation of experts in every case.

#### ***LDL***

Similar to SBP and DBP, for LDL manual and automatic classifications were consistent.

#### ***Total cholesterol risk***

Some patient risk classifications differed between our automated analysis and the expert annotations. In each case, the risk score fell between 5 and 5.2. Interestingly, in the American Heart Association guidelines (Table 4) there is a gap in their measurement-continuum, resulting in a lack of any interpretation-guidance for measurements between 5 and 5.2. Our automated analysis therefore revealed that the clinical expert had compensated for this gap by assigning these measurements to the "low risk" category. By modifying our OWL model to change the low risk cut-off level from 5 to 5.2, we were then able to achieve perfect correspondence with the clinical expert; moreover, this correspondence shows that the clinician had used this 5.2 boundary as their upper limit for low-risk when undertaking their binary classification.

Original AHA guideline in OWL:

HighRiskCholesterolRecord=

PatientRecord ***and***

(sio:hasAttribute ***some***

(cardio:SerumCholesterolConcentration ***and***

sio:hasMeasurement ***some*** ( sio:Measurement and

(sio:hasUnit ***value*** cardio:mili-mole-per-liter) ***and***

(sio:hasValue ***some*** double[> = 5.0]))))

LowRiskCholesterolRecord=

PatientRecord ***and***

(sio:hasAttribute ***some***

(cardio: SerumCholesterolConcentration ***and***

sio:hasMeasurement ***some*** ( sio:Measurement and

(sio:hasUnit ***value*** cardio:mili-mole-per-liter) ***and***

(sio:hasValue ***some*** double[< 5.0]))))

Modified model:

HighRiskCholesterolRecord=

PatientRecord ***and***

(sio:hasAttribute ***some***

(cardio:SerumCholesterolConcentration ***and***

sio:hasMeasurement ***some*** ( sio:Measurement and

(sio:hasUnit ***value*** cardio:mili-mole-per-liter) ***and***

(sio:hasValue ***some*** double[> = 5.2]))))

LowRiskCholesterolRecord=

PatientRecord ***and***

(sio:hasAttribute ***some***

(cardio: SerumCholesterolConcentration ***and***

sio:hasMeasurement ***some*** ( sio:Measurement and

(sio:hasUnit ***value*** cardio:mili-mole-per-liter) ***and***

(sio:hasValue ***some*** double[< 5.2]))))

#### ***HDL and Triglyceride risk***

Having no guidance on how to build the model in these cases where the clinical (binary) classification had no correspondence to the three or four level categorization system of the official guidelines, we first modeled the extreme cases (high/low, ignoring borderline/medium categories), expecting to find complete congruence with the expert annotation at least for these patients. Surprisingly, in neither case did our automated categorization match the expert clinical categorization. We determined (by manual inspection) that in these cases the clinician did not follow any of the guideline category boundaries for their binary classification rather, they "invented" boundaries reflecting their personal opinion of risk. In the case of HDL, the boundary was well under the official lower limit (0.89 mmol/L compared to the official boundary of 1.03 mmol/L), whereas for Triglyceride measurements the clinician chose a cutoff between the guidelines range for "High" risk (2.26-5.65 mmol/L). The original OWL models, and the adjusted OWL models are shown below. The adjusted models provided perfect correspondence with the expert clinical classification when used in our automated framework.

#### ***HDL***

Original AHA guideline in OWL:

HighRiskHDLCholesterolRecord=

PatientRecord ***and***

(sio:hasAttribute ***some***

(cardio:SerumHDLCholesterolConcentration ***and***

sio:hasMeasurement ***some*** ( sio:Measurement and

(sio:hasUnit ***value*** cardio:mili-mole-per-liter) ***and***

(sio:hasValue ***some*** double[<= 1.03]))))

LowRiskHDLCholesterolRecord=

PatientRecord ***and***

(sio:hasAttribute ***some***

(cardio: SerumCholesterolConcentration ***and***

sio:hasMeasurement ***some*** ( sio:Measurement and

(sio:hasUnit ***value*** cardio:mili-mole-per-liter) ***and***

(sio:hasValue ***some*** double[> 1.55]))))

Modified model:

HighRiskHDLCholesterolRecord=

PatientRecord ***and***

(sio:hasAttribute ***some***

(cardio:SerumHDLCholesterolConcentration ***and***

sio:hasMeasurement ***some*** ( sio:Measurement and

(sio:hasUnit ***value*** cardio:mili-mole-per-liter) ***and***

(sio:hasValue ***some*** double[<= 0.89]))))

LowRiskHDLCholesterolRecord=

PatientRecord ***and***

(sio:hasAttribute ***some***

(cardio: SerumCholesterolConcentration ***and***

sio:hasMeasurement ***some*** ( sio:Measurement and

(sio:hasUnit ***value*** cardio:mili-mole-per-liter) ***and***

(sio:hasValue ***some*** double[> 0.89]))))

#### ***Triglyceride***

Original AHA guideline in OWL:

HighRiskTriglycerideRecord=

PatientRecord ***and***

(sio:hasAttribute ***some***

(cardio:SerumTriglycerideCholesterolConcentration ***and***

sio:hasMeasurement ***some*** ( sio:Measurement and

(sio:hasUnit ***value*** cardio:mili-mole-per-liter) ***and***

(sio:hasValue ***some*** double[> = 2.26]))))

LowRiskTriglycerideRecord=

PatientRecord ***and***

(sio:hasAttribute ***some***

(cardio: SerumTriglycerideConcentration ***and***

sio:hasMeasurement ***some*** ( sio:Measurement and

(sio:hasUnit ***value*** cardio:mili-mole-per-liter) ***and***

(sio:hasValue ***some*** double[<1.69 ]))))

Modified model:

HighRiskTriglycerideRecord=

PatientRecord ***and***

(sio:hasAttribute ***some***

(cardio:SerumTriglycerideCholesterolConcentration ***and***

sio:hasMeasurement ***some*** ( sio:Measurement and

(sio:hasUnit ***value*** cardio:mili-mole-per-liter) ***and***

(sio:hasValue ***some*** double[> = 2.63]))))

LowRiskTriglycerideRecord=

PatientRecord ***and***

(sio:hasAttribute ***some***

(cardio: SerumTriglycerideConcentration ***and***

sio:hasMeasurement ***some*** ( sio:Measurement and

(sio:hasUnit ***value*** cardio:mili-mole-per-liter) ***and***

(sio:hasValue ***some*** double[<2.63 ]))))

#### ***Body Mass Index risk***

Similarly, we determined from our results that the guideline used by the expert in their classification was more relaxed than the AHA guidelines. By changing the threshold in our OWL class definition from 25 to 26, we were able to achieve perfect correspondence with the expert’s annotations. It is important to point-out, with respect to this measurement, that we did not need to modify the analytical Web Service in order to achieve this correspondence - only the OWL model needed to be adapted to match the interpretation of the clinical expert. The significance of this observation will be discussed later.

Original AHA guideline in OWL:

HighRiskBMIRecord=

PatientRecord ***and***

(sio:hasAttribute ***some***

(cardio:BodyMassIndex ***and***

sio:hasMeasurement ***some*** ( sio:Measurement and

(sio:hasUnit ***value*** cardio:kilogram-per-meter-squared) ***and***

(sio:hasValue ***some*** double[> = 25.0]))))

LowRiskBMIRecord=

PatientRecord ***and***

(sio:hasAttribute ***some***

(cardio:BodyMassIndex ***and***

sio:hasMeasurement ***some*** ( sio:Measurement and

(sio:hasUnit ***value*** cardio:kilogram-per-meter-squared) ***and***

(sio:hasValue ***some*** double[< 25.0]))))

Modified model:

HighRiskBMIRecord=

PatientRecord ***and***

(sio:hasAttribute ***some***

(cardio:BodyMassIndex ***and***

sio:hasMeasurement ***some*** ( sio:Measurement and

(sio:hasUnit ***value*** cardio:kilogram-per-meter-squared) ***and***

(sio:hasValue ***some*** double[> = 26.0]))))

LowRiskBMIRecord=

PatientRecord ***and***

(sio:hasAttribute ***some***

(cardio:BodyMassIndex ***and***

sio:hasMeasurement ***some*** (sio:Measurement and

(sio:hasUnit ***value*** cardio:kilogram-per-meter-squared) ***and***

(sio:hasValue ***some*** double[< 26.0]))))

#### ***Evaluation of automated ternary risk classification***

**Figure 5:**
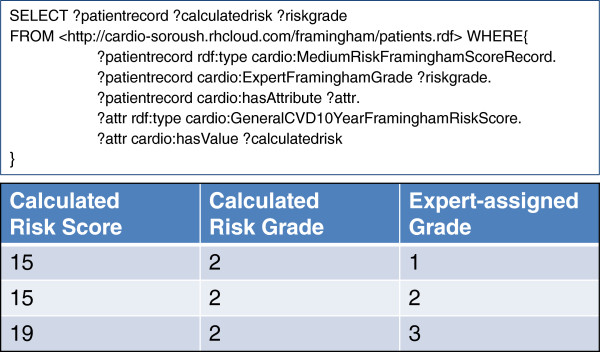
SPARQL queries and a small snapshot of the results for automatic classification of patients into “high risk”, “medium risk” and “low risk”, respectively.

Figure 5 shows the SPARQL query which automatically classifies patient records into the "moderate risk" Framingham guidelines OWL model, compared with the annotations done manually by experts (see supplementary material [27] for other Framingham guideline query/result pairs). Below this is an abbreviated table of exemplar query output specifically showing rows of discrepancy which are of particular interest for discussion.

### **Discrepancies between automated and expert ternary classifications**

#### ***Framinghamrisk***

No expert-annotated “high risk record” was classified as “low risk record” by automatic classification or vice versa only the “moderate risk records” were differentially-classified by our automated approach compared to the clinical expert classification. Interestingly, however, the automated interpretations included both higher- and lower-risk classifications compared to the expert annotations. As can be seen in the first two rows of the Figure 5 results table, the same calculated Framingham risk-score of 15 was classified as being “low risk” and “medium risk” respectively by the expert clinician, while another "medium risk" score (19) was classified as "high risk" by the expert. After trouble-shooting the code and the ontological definitions, we examined the scores to determine if, as with the binary classifications above, it would be possible to improve our performance by relaxing or tightening certain constraints in the OWL class definitions, however we determined that this was not possible. This suggests that other factors, not captured by the guidelines, have led the clinical expert to select one or the other risk category for any given patient. In discussions with the clinician, we learned that the patients were under varying regimes of pharmaceutical blood pressure treatment, and that this would have affected their risk-assessment. We are undertaking a follow-up study in which we attempt to semantically model and add drug treatment regimes to the patient's profiles and our risk models to determine if this is sufficient to resolve all cases of mis-classification by our automated system, or if there remain yet additional factors that are being used by clinicians to make their risk assessment. Regardless, we may not ever be able to determine, with any certainty, the bases for the original risk classifications, and this is an important point for discussion.

## **Discussion**

### **Interpreting discrepancies between automated and manual risk classification**

It is first important to note that the data in our study - in particular, the risk classifications of the patients - were not used for the purpose of selecting an intervention in the course of the patient's clinical care. We presumed that clinical researchers would use existing published guidelines for categorization in the course of their clinical research, but this was not necessarily a valid premise.

In our results, we note a variety of discrepancies between our initial OWL models' rigorous adherence to published clinical standards, and the evaluation and phenotypic classification by the expert clinical researcher. Some of these were due to missing data in the guidelines themselves, where we were able to, with reasonable confidence, guess what the clinician's interpretation of the guideline was and model that interpretation. Others were due to the researcher "bending" the guidelines either to match their personal beliefs, or because it was more appropriate for the research question they were asking. We were similarly able to (as far as we can tell) accurately modify the OWL models to match the clinician's expert opinion in many of these cases. Some cases, however, have so far eluded our ability to capture, in OWL, what the intent or rationale of the researcher was. Nevertheless, assuming that the decisions are not "arbitrary", we are confident that with further study we will be able to construct OWL models that correspond to these complex clinical interpretations. Moreover, while in this pilot study, we manually modified the cutoff levels of the OWL models after visual inspection of the data, our subsequent studies undertake to determine these boundaries using data-mining and pattern-detection approaches, thus this should not be considered an insurmountable weakness of the current work.

That experts (at least in our case, but we believe it is likely to be true for many cases) do not strictly follow published guidelines when classifying patients in their clinical research is, in itself, not surprising; however, it does have implications for both reproducibility of clinical studies, as well as the accuracy and interpretation of statistically sensitive high-throughput studies such as GWAS. Potential factors that influence experts to deviate from guidelines may include clinical observations outside of those that make up the guideline, or other non-clinical yet measurable/detectable features. Regardless, it is important for reproducibility and rigor that experimental methods be fully explained and detailed, yet at the same time it is undesirable (in fact, likely impossible) to force clinical researchers to follow guidelines which go against their expert beliefs. As such, a middle-ground is needed where experts retain their "personalized" classification system, and yet have this system formally encoded in a transparent, publishable, and re-usable manner.

In this study, we demonstrate that the semantic modeling approach we advocate here provides re-usable, rigorous models which are nevertheless flexible, allowing individual, personalized expert-knowledge to be encoded, published, and shared. Moreover, these rigorous yet personalized ontological models can be used to drive the automated analysis of data, removing the individual from the analytical process. This is important because "analysis tweaking", based in human intervention in the analytical or interpretative process, historically has gone unrecorded and thus led to non-reproducible science. Our approach, while not preventing the expert from imposing their own interpretation on the data (in fact, *encouraging* it!), ensures that in order to "tweak" the analysis, such an intervention must be made explicit in their ontological model; moreover, the resulting ontology can be published together with the study results to ensure transparency. Not only does this facilitate reproducibility of the study by making the personal expert opinion/interpretation accessible to other researchers, but it also allows explicit and accurate comparison between the formally-encoded expert opinions of a diverse community of clinical researchers, and the ability to use a third-party interpretation to investigate your own data - i.e. the ability to "see your data through the eyes of another". We think this is a powerful new approach to transparent and reproducible clinical research, where ideas and interpretation-regimes are explicitly recorded, shared, and compared.

### **Broader implications of "personalizing" OWL ontologies**

Our use of OWL in this study differs markedly from the norm in the biomedical community, where ontologies are used primarily to compel harmonization around a particular world view, thus facilitating cross-study comparisons by *dis*-allowing individual opinion or interpretation. In contrast, we began from the perspective that individual clinicial researchers would insist upon their authority, as experts, to classify patients in whatever way they thought was correct for a particular study, and would resist forced adherence to guidelines (in fact, in some cases it is the guidelines themselves that are the topics of investigation and evaluation). Indeed, we demonstrate in this study that clinicians frequently deviate from established clinical guidelines, yet we also demonstrate that OWL classes can be constructed to model an individual clinician's expert perspective, thereby making their interpretation transparent, and re-usable in a rigorous manner. Most importantly, however, our inability in some cases to accurately reproduce the interpretation of the expert *post facto*, even after manually re-modeling the guidelines, shows the danger of *not* capturing these personal, expert perspectives in some formal framework such as OWL at the time the experiment is being run. These ontologies, representing individual perspectives on how data should be interpreted, resemble *in silico* hypotheses - the belief system of the individual undertaking the study, which may or may not be correct and/or shared by any other researcher. In this study, we demonstrate that these clinical hypotheses can be automatically evaluated over real patient data using existing Semantic Web tools and frameworks.

## **Conclusions**

This study had several, largely methodological, objectives. First, there are a large number of "legacy" datasets that would be of benefit to researchers if they were published on the Semantic Web. We demonstrated a workable path for conversion and publication of these datasets that provided advantages beyond simply making the data available as "triples", but also in making it semantically transparent such that it could be easily re-analyzed by third-party researchers using their own classification frameworks. Second, the majority of ontologies available in the life sciences to date are class hierarchies, where the labels of each class are largely used to standardize annotations. The ability to logically reason over these labels is quite limited, thus inhibiting their use for automated annotation and classification of data. Nevertheless, these ontologies are increasingly comprehensive and reflect expert consensus of what concepts are relevant in a given domain. Here, we proposed and demonstrated a path for extending an existing ontology such that it could be utilized by DL reasoners to dynamically classify and interpret datasets - a process that is currently done largely by experts. Third, we demonstrated that clinical phenotype classification systems could be modeled in the OWL language by taking advantage of the rich, axiomatic structure of OWL-DL ontologies, and a variety of analytical Web Services. We showed how this combination of ontologies and Services can be used to make clinical data analyses both more transparent and more automated. Finally, we showed that individual clinicians deviate from established clinical guidelines at every layer of an analysis, and this demonstrates the need for a formal, yet personalized clinical interpretation framework to ensure transparency and reproducibility. We demonstrate that this can be achieved by creating and publishing "personalized" OWL ontologies.

## Endnotes

^a^It should be noted that even though ‘kilogram” is considered a base unit in SI, it is composed of kilo prefix plus base unit “gram”. In this sense, kilogram is an exception of a unit which is considered as base unit in SI system. However, to stick to our design schema, we considered “gram” as the base and defined kilogram as an extension of it.

## **Abbreviations**

AHA: American heart association; BMI: Body mass index; CABG: Coronary allograft bypass graft; DBP: Diastolic blood pressure; G: Gram; HDL: High-density lipoprotein; Kg: Kilogram; kg/m2: Kilogram per meter squared; KPa: Kilopascal; LDL: Low-density lipoprotein; M: Meter; mmHg: Millimeter of mercury column; OWL-DL: Web ontology language - description logic; Pa: Pascal; PATO: Phenotypic quality ontology; PCI: Percutaneous coronary intervention; RDF: Resource description framework; SADI: Semantic automated discovery and integration; SBP: Systolic blood pressure; SIO: SemanticScience integrated ontology; TG: Triglyceride.

## **Competing interests**

The authors have no competing interests to declare.

## **Authors' contributions**

SS and MW planned this work, and jointly wrote this manuscript. SS executed the data migration, ontology design and extension, web service deployment, and overall analysis. BM generated and initially analyzed the source clinical data-set, and discussed and validated our approach and choice of clinical standards. All authors have read, revised and approved this manuscript.

## References

[B1] RDF Semantic Web Standards http://www.w3.org/RDF/

[B2] OWL Web Ontology Language Overview http://www.w3.org/TR/owlfeatures/

[B3] Zakkar M Hornick P Surgery for coronary artery disease Surgery (Oxford) 2007 25 5 231 37 10.1016/j.mpsur.2007.04.007

[B4] Chobanian AV Bakris GL Black HR Black HR Cushman WC Green LA Izzo JL Jr Jones DW, Materson BJ, Oparil S, Wright JT Jr, Roccella EJ: Seventh Report of the Joint National Committee on Prevention, Detection, Evaluation, and Treatment of High Blood Pressure JAMA 2003 290 2 197 10.1161/01.HYP.0000107251.49515.c2 14656957

[B5] Global Initiative for Chronic Obstructive Lung Disease http://www.who.int/respiratory/copd/GOLD_WR_06.pdf

[B6] OpenGALEN Mission Statementhttp://www.opengalen.org/index.html

[B7] Dawber TR Moore FE Mann GV Coronary heart disease in the Framingham study Am J Public Health 1957 47 3 4 24 10.2105/ajph.47.4_pt_2.4 PMC1550985 13411327

[B8] Ridker PM Buring JE Rifai N Cook NR Development and validation of improved algorithms for the assessment of global cardiovascular risk in women: the Reynolds Risk Score JAMA 2007 297 6 611 619 10.1001/jama.297.6.611 17299196

[B9] BioMOBY Interoperability Today, Integration Tomorrow http://sadiframework.org/documentation/MOBY_IMB_UQ2005.ppt

[B10] Wilkinson MD Vandervalk B McCarthy L The Semantic Automated Discovery and Integration (SADI) Web service Design-Pattern, API and Reference Implementation J Biomed Semantics 2011 2 8 10.1186/2041-1480-2-8 22024447 PMC3212890

[B11] Vandervalk B McCarthy L Wilkinson MD SHARE: A Semantic Web Query Engine for Bioinformatics The Semantic Web, Lecture Notes in Computer Science proceedings of the ASWC 2009 5926 367 369 10.1007/978-3-642-10871-6_27

[B12] DOLCE units of measurements http://www.w3.org/2001/sw/BestPractices/WNET/DLP3941_daml.html#measurement-unit%20#5

[B13] Claudio Masolo Stefano Borgo Qualities in Formal Ontology Foundational Aspects of Ontologies (FOnt 2005) Workshop 2005 Trento, Italy 2 16

[B14] Measurement Unit Ontology http://forge.morfeoproject.org/wiki_en/index.php/Units_of_measurement_ontology#Measurement_Units_Ontology_.28MUO.29

[B15] International System of Units. 8th editionhttp://www.bipm.org/utils/common/pdf/si_brochure_8_en.pdf

[B16] Garabed Eknoyan Adolphe Quetelet (1796–1874)—the average man and indices of obesity Nephrol Dial Transplant 2008 23 1 47 51 17890752 10.1093/ndt/gfm517

[B17] School of Computer Sciencehttp://www.cs.manchester.ac.uk/

[B18] A brief historyhttp://www.opengalen.org/tutorials/grail/tutorial21.html

[B19] Isabel Cruz The semantic web - ISWC 2006 5thInternational Semantic Web Conference 2006 GA, USA: SWC 2006 Athens 664

[B20] Dumontier M Semanticscience Integrated Ontology (SIO) [http://code.google.com/p/semanticscience/wiki/SIO] 10.1186/2041-1480-5-14 PMC4015691 24602174

[B21] Why and how SIO differs from OBO Foundry efforthttp://groups.google.com/group/sio-ontology/browse_thread/thread/e5aa67843aaad402?pli=1

[B22] Gruber TR A translational approach to portable ontologies specifications Knowledge Acquisition 1993 5 2 199 220 10.1006/knac.1993.1008

[B23] Dumontier M Villanueva-Rosales N Modeling Life Science Knowledge with OWL 1.1 2008 OWL Experiences and Design (OWLED-DC, Washington DC, USA

[B24] Units of Measurementshttp://bioportal.bioontology.org/ontologies/45500?p=terms

[B25] Phenotypic Quality Ontologyhttp://obofoundry.org/cgi-bin/detail.cgi?quality

[B26] Friedewald WT Levy RI Fredrickson DS Estimation of the concentration of low-density lipoprotein cholesterol in plasma, without use of the preparative ultracentrifuge Clinical Chemistry 1972 18 6 499 502 4337382

[B27] Supplementary Datahttp://cardio-soroush.rhcloud.com/framingham/supplementary.pptx

[B28] What cholesterol levels mean http://www.heart.org/HEARTORG/Conditions/Cholesterol/AboutCholesterol/What-Your-Cholesterol-Levels-Mean_UCM_305562_Article.jsp]

[B29] Cholesterol Levelshttp://lightup-ym.com/2011/04/10/the-cholesterol-levels/

[B30] Body Mass Index (BMI) http://www.heart.org/HEARTORG/GettingHealthy/WeightManagement/BodyMassIndex/Body-Mass-Index-BMI-Calculator_UCM_307849_Article.jsp

[B31] Framingham Heart Studyhttp://www.framinghamheartstudy.org/risk/index.html

